# Deciphering growth abilities of *fusarium oxysporum* f. sp. *pisi* under variable temperature, pH and nitrogen

**DOI:** 10.3389/fmicb.2023.1228442

**Published:** 2023-08-03

**Authors:** Kota Chakrapani, W. Tampakleima Chanu, Bireswar Sinha, Bijeeta Thangjam, Wajid Hasan, Konjengbam Sarda Devi, Tusi Chakma, Sumitra Phurailatpam, Lokesh Kumar Mishra, Gopi Mohan Singh, Pramesh Khoyumthem, Rahul Saini

**Affiliations:** ^1^Department of Plant Pathology, College of Agriculture, Central Agricultural University, Imphal, India; ^2^KVK, Jahanabad, Bihar Agricultural University, Jahanabad, India; ^3^Department of BPME, College of Agriculture, Central Agricultural University, Imphal, India; ^4^Department of Agricultural Statistics, College of Agriculture, Central Agricultural University, Imphal, India; ^5^Department of Genetics and Plant Breeding, AICRP (Groundnut), College of Agriculture, Central Agricultural University, Imphal, India; ^6^Department of Entomology, College of Agriculture, Chaudhary Charan Singh Haryana Agricultural University, Hisar, India

**Keywords:** wilt, *Fusarium oxysporum* (Fop) isolates, climate change, growth, temperature, pH, nitrogen dose

## Abstract

Fusarium wilt caused by *Fusarium oxysporum* f. sp. *pisi* (*Fop*) is an important disease and major obstacle to pea production, causing huge losses to growers. The focus of this study was on isolation followed by morphological, molecular characterization and analyzing the growth of the casual agent under variable temperature, pH and Nitrogen levels. The morphological features of radial growth, sporulation, pigmentation and mycelial characterization were examined and the variability of all isolates was presented. Molecular characterization of the fungus by ITS rDNA sequencing revealed that all 13 isolates belong to *Fusarium oxysporum* species. Six isolates were tested for temperature, pH and nitrogen dosage optimization studies. Seven different temperatures, viz., 21, 23, 25, 27, 29, 31, 33°C and pH values, having 3, 4, 5, 6, 7, 8, and 9 pH, as well as nitrogen dosage levels of 0 g, 3 g, 5 g, 7 g, 9 g, 11 g, and 13 g were tested against all six isolates, respectively. The results showed that all isolates exhibited the highest growth at a temperature of 25°C and the optimal temperature range for growth of *Fusarium oxysporum* was 23–27°C. All isolates showed the highest growth at pH5. Change in the nitrogen doses of the base ended in formation of thick, dense, fluffy mycelium of the casual agent. Six isolates were used for combination studies with seven different levels of temperatures, pH levels and nitrogen dosages. The density plots revealed the variations in the growth of the isolates with changes in temperature, pH and nitrogen levels, which can lead to mutations or genetic changes in the pathogens that could potentially introduce new threats to pea cultivation.

## Introduction

Garden pea (*Pisum sativum* L.) is a popular winter legume commonly used as food for humans and animal feed. Pea seeds are a rich source of carbohydrates (56–74%), proteins (21–33%), and minerals like iron (97%), selenium (42%), molybdenum (12%), and zinc (41%) (Parihar et al., 2020, 2021, 2022). All of these nutrient components in peas are a key ingredient for national food security that can be fed to people. In addition, pea seeds are able to minimize the risk of various chronic diseases such as diabetes, maintain blood cholesterol levels, and promote cardiovascular health as they have significant amounts of antioxidants, Vitamin (A, B, E, and K), omega fatty acids and anti-inflammatory agents (Ekvall et al., 2006; Marinangeli and Jones, 2011; Singh et al., 2013). Further, the role of Garden pea as an important crop rotation promoting sustainable cropping system due to their inherent ability to fix nitrogen leading to balanced soil health and productivity is widely acknowledged (Sharma et al., 2020). According to the Food and Agriculture Organization (FAO, Food and Agriculture Organization, 2020), the global statistics for green pea and dry pea production in 2020 are 49.7 megatons and 47.2 megatons, respectively. The Indian subcontinent contributes to the bulk of pea production globally and ranks second to China in green pea production at 5.73 mega tonnes. In terms of dried pea production, India ranks fifth after Russia with 0.8 mega tonnes. It is estimated that the world population could reach 10 billion by 2050 and the production of nutritious food should be ensured to continue to feed the growing population despite the reduction in arable land. In addition, increasing climate changes and already vulnerable industrial systems are causing severe stress on farmland, public health, and the ecosystem (Anonymous, 2019).

The major barricade in hampering Garden pea production is biotic stress with the soil borne diseases being the most notable factor posing threat to pea farming across the world. Fusarium wilt (FW) caused by *Fusarium oxysporum* f. sp. *pisi* (*Fop*) is one of the major biotic stress that leads to continuous and severe yield losses. Buxton and Storey (1954) reported the emergence of pea wilt in Britain as a serious problem and found an association with Fusarium species. *Fop* is a soil-borne fungus that includes both non-pathogenic strains and economically important plant pathogens. It has been known in the United States since 1928. In India, the disease was first reported by Sukapure et al. (1957) from Bombay. Chupps and Sherf (1960) first recognized Fusarium wilt of peas in Minnesota in 1918. Pea wilt disease was first elucidated and distinguished from root rot by Jones and Linford (1925). They designated it as undescribed wilt disease. The disease caught on and was found in 50 fields in Wisconsin, causing huge losses to pea growers in the area compared to root rot disease. The causative agent of the disease was named as Fothoceras App and Wr var. *pisi* in 1928 (Linford, 1928). Over time, the name race 1 of *Fusarium oxysporum* Schl f.sp. Due to the characterization, *pisi* (van Hall) was named as the causal factor. Snyder and Hansen (1940) reported that the genus *Fusarium* belongs to the class Fungi *imperfecti*, which includes many species and many forms within species. Based on host interactions, Kraft (1994) classified numerous isolates of *Fusarium oxysporum* f.sp. *pisi* and found that it is controlled by the genetic makeup of both the host and the pathogen. The fungus protrudes the root tissue and invades the xylem vessels of the vascular bundles, clogging the bundles and restricting the movement of water minerals to the top of the plant. This leads to yellowing and drying symptoms of the infected plant. If the invasion occurs in earlier stages of the plant, the fungus will kill the plant. When invasion occurs at later stages, kernel shrinkage and loss of high yields can be visualized (Linford, 1928). The fungus enters plant tissues either through wounds or by direct penetration of the epidermis (Nyvall and Haglund, 1972). Armstrong and Armstrong (1974) revealed their findings that when the fungus invades wounds, the frequency of symptom onset and the rate of wilting increase. *Fusarium solani* f.sp. *pisi* and *Fusarium oxysporum* f.sp. *pisi* infects the root, invades the xylem vessels and becomes associated with the plants and seeds (Maheshwari et al., 1980). Lin (1991) reported that wilt caused by *Fusarium oxysporum* f.sp. *pisi* that appear in the flowering phase can be accompanied by yellowing of the leaves and discoloration.

The yield-limiting factor in form of “*Fop*” can result in 100% loss of crop (Aslam et al., 2019; El-Sharkawy et al., 2021; Deng et al., 2022). When the pea crop is included in crop rotation, it paves the way for *Fop* to build up the inoculum in the soil. The extraordinary nature of chlamydospores ensures that the pathogen lives and is viable in the soil for more than 10 years (Kraft et al., 1994; Aslam et al., 2019; El-Sharkawy et al., 2021; Deng et al., 2022). Previous records show that four races of *Fop* have been identified and characterized as races 1, 2, 5, and 6, respectively (Haglund and Kraft, 1970, 1979; Jenkins et al., 2021). Strains demonstrate resistance to *Fop* by having a single dominant gene (one for each race) for races 1, 5, and 6, whereas race 2 resistance has been identified as quantitative, exaggerating the severity (McPhee et al., 2012; Bani et al., 2018). To deal with the consequences, the focus should be on researching intelligent technologies in agriculture and adapting the country to the climate crisis and food security. In this context, we want to better understand the pathogen and its response to harsh climatic conditions. Our aim is to study the optimization conditions and combination effects of climatic factors on the growth of *Fop* isolates.

Being a part of Disease cycle, Abiotic factors, i.e., environmental factors, play a key role in disease incidence and severity. Evidence on interaction between temperature, pH and nitrogen levels on radial growth of the *Fusarium* spp. has been reported (Jones, 1924; Chen et al., 2013). Temperature has the ability of altering the expression of host defence mechanisms that favors resistance or susceptible. Banana, chickpea, carnation and lettuce had significant changes in their host defence mechanism from moderately resistant to susceptible when inoculated with their respective *Fusarium* spp. when the temperature raised above ~22–28°C. (Harling et al., 1988; Brake et al., 1995; Landa et al., 2006; Stuthman et al., 2007; Scott et al., 2010). Similarly pH of the soil has the ability to alter *Fusarium* spp. with respect to growth, spore adherence to plant roots ad appearance of wilt symptoms on host exposed to different temperatures (Chen et al., 2013; Gatch and du Toit, 2017).

## Materials and methods

### Pathogen isolation and characterization

The pea plants showing typical wilting symptoms were collected from the main pea growing areas of Manipur. The infected plant roots were examined for vessel discoloration. The roots, showing the typical vessel discoloration, were rinsed with tap water and torn into 4–5 mm sections. The sections were then subjected to surface sterilization with 2% sodium hypochlorite solution for 1 min. The sections were then transferred to sterile distilled water to delineate traces of sodium hypochlorite. Washing with sterile distilled water was repeated three times to ensure tissue sections were free of sterilizing chemicals. The sterilized tissues were blotted with blotting paper to remove the excess water. Using sterile forceps, sections were plated onto water agar plates and incubated at 25 ± 1°C for 48 to 72 h. Colonies resembling *Fusarium oxysporum* were subcultured using the hyphal tip culture method to obtain the pure form of the isolate. The pure cultures were stored at −20°C for future use.

Morphological characterization of the isolates was done by inverting 5 mm disc of 7 days old culture on PDA plates. Sporulation studies carried out by taking 5 mm disc of the culture plate and placing the disc in 10 mL sterile distilled water. The spores were dislodged into water by continuous shaking. A drop of this spore suspension was placed on to a haemocytometer/ Neubauer Chamber and the spore count was taken from 5 squares at random. The spore count per ml was calculated by using a formula given by Pathak (1984).


$$\;\mathrm{Number of spores}\;\mathrm{per}\;\mathrm{mL}=\frac{N*1000}{X}$$

where:

*N* = Total No. of spores counted/No. of squares,

*X* = Volume of mounting solution between the cover glass and above the squares counted.

The spore morphology and size was determined by placing a drop of spore suspension of each isolate onto a clean glass slide and covered with a cover slip. The slides were observed under 40X magnification of the microscope (Olympus BX41). Size of the sopre was determined by using ocular and stage micrometers with the help of Biowizard 4.2 image analyzer.

All isolates were pure and were subcultured onto PDA plates for 7 days. The 7-day-old cultures were then subcultured in PDB containing no agar to obtain a mycelial mat. The mycelial mat was collected after 7 days of incubation. Then the mycelial mats of each isolate were harvested for extraction of total genomic DNA using HiPure ATM Fungal DNA Purification Kit (Hi Media, India). The genomic DNA was extracted according to the manufacturer’s protocol. PCR amplifications were performed using primers ITS-1 (5 TCC GTA GGT GAA CCT GCC G 3) and ITS-4 (5 TCC TCC GCT TAT TGA TAT GC 3). The PCR products obtained by amplification with universal primers targeting the rRNA gene were sent to Xcelris Labs Ltd., Ahmadabad, Gujarat, India for sequencing using the same upstream and downstream primers. The sequenced data were analyzed in NCBI BLAST to identify the species of all isolates.

### Optimization studies to evaluate the growth of different *Fop* isolates at different temperatures and different pH levels

The culture media PDA was prepared according to standard protocols. Seven different temperatures viz*.,* 21, 23, 25, 27, 29, 31 and 33°C were used for optimization studies in which the growth of six *Fop* isolates was compared and analyzed. The culture media were then subjected to sterilization according to sterilization standards. The media were dispensed into petri dishes in an aseptic environment. The petri dishes were then inoculated with 5 mm pieces of 7 day old cultures of each isolate. The inoculated petri dishes were subjected to incubation in BOD at their respective temperatures. Growth of the isolates was noted after 7 days of inoculation. Three replicates were performed for each isolate at each temperature. Similarly, the PDA, culture medium was prepared following the standard protocols. Seven different pH values viz*.,* 3, 4, 5, 6, 7, 8, and 9 were used for the optimization studies, in which the growth of six *Fop* isolates was compared and analyzed. The pH of the culture medium was changed by 0.1 N hydrochloric acid and 0.1 N sodium hydroxide with the digital pH meter (EuTech Instruments, pH700, Thermo Fisher, Scientific, United States). The culture medium was then subjected to sterilization according to sterilization standards. The medium was delivered to Petriplates under aseptic environment. The petri dishes were then inoculated with 5 mm pieces of 7 day old cultures of each isolate. The inoculated Petri dishes were subjected to incubation in BOD at a temperature of 25 + 1°C. The growth of the isolates was noted after 7 days of inoculation. Three replicates for each isolate at each pH were maintained.

### Combination effect of temperature, pH and nitrogen dose levels on radial growth on six isolates of *Fop*

To study the combined effect of different pH and different temperature levels on the growth of *Fop* isolates, a pH range of 3–9, i.e., 3, 4, 5, 6, 7, 8, and 9, and a temperature range of 21–33°C, i.e., 21, 23, 25, 27, 29, 31, 33°C and nitrogen levels of 0–13 g, i.e., 0, 3.5, 7, 9, 11, and 13 g each were used in different combinations. The pH and temperature range was calibrated using the optimal pH and temperature, determined from previous studies. To assess radial growth study of each isolate, culture medium PDA was prepared according to standard protocols. The pH of the culture medium was changed by 0.1 N hydrochloric acid and 0.1 N sodium hydroxide with the digital pH meter used (EuTech Instruments, pH700, Thermo Fisher, Scientific, United States). The culture media of individual pH was prepared separately. The culture media were then subjected to sterilization according to sterilization standards. The media were dispensed into petri dishes in an aseptic environment. The petri dishes were then inoculated with 5 mm pieces of 7 day old cultures of each isolate. The inoculated petri dishes were subjected to incubation in BOD at specific temperatures, i.e., 21, 23, 25, 27, 29, 31, and 33°C. Growth of the isolates was noted after 7 days of inoculation. Three replicates were performed for each isolate at each pH and temperature. Similarly, the culture medium PDA was prepared, to which a nitrogen source (peptone) was added as per the requisite viz., peptone powder (RM001-Hi media) was weighed accordingly using a weighing balance (Precession weighing balance KERN 572) i.e., 3. 5, 7, 9, 11, and 13 g and was suspended in a litre of PDA. The prepared media was subjected for sterilization. The isolates were inoculated according to the protocol mentioned in the above step. Three replications were performed for each isolate independently.

### Data analysis

Completely randomized design and a three-factorial randomized block design were used to analyze the data generated. For the sake of simplicity, the means of the raw data have been presented in the form of numbers. However, R Studio was used to analyze the data and create density plots. The significance of the differences between the means was determined by mean comparison tests.

## Results

### Pathogen isolation and characterization

A total of 13 isolates were isolated from samples showing typical wilt symptoms collected from different locations of Manipur. Isolation was performed at the Department of Plant Pathology, College of Agriculture, Central Agricultural University, Imphal. The isolates were designated *Fop*-1, *Fop*-2, *Fop*-3, *Fop*-4, *Fop*-5, *Fop*-6, *Fop*-7, *Fop*-8, *Fop*-9, *Fop*-10, *Fop*-11, *Fop* – 12 or *Fop*-13. The details of the isolates collected are presented in Table 1.

**Table 1:** Location specificities of samples collected for isolation of the casual pathogen.

Sl. No.	Isolates	Place/Site	Latitude (N)	Longitude (E)	Altitude (ft)
1	*Fop*-1	Kongba	24° 46.071’	93° 57.578’	2,533
2	*Fop*-2	Wangjing Lamding	24° 36.159’	94° 02.206’	2,525
3	*Fop*-3	Kangla Sangomshang	24° 50.859’	93° 59.531’	2,566
4	*Fop*-4	Hiyangthang	24° 40.314’	93° 55.021’	2,536
5	*Fop*-5	Itam Nungoi	24° 52.202’	94° 01.674’	2,579
6	*Fop*-6	Toubul	24° 37.638’	93° 46.068’	2,584
7	*Fop*-7	Iramsiphei Mayai Leikai	24° 39.779’	93° 55.605’	2,534
8	*Fop*-8	Awangkhul	24° 48.806’	93° 51.863’	2,549
9	*Fop*-9	Nambol Phoijing	24° 43.783’	93° 50.764’	2,508
10	*Fop*-10	Lungphou Dam	24° 35.528’	94° 01.233’	2,516
11	*Fop*-11	Lamding Khumnthem	24° 35.528’	94° 01.485’	2,529
12	*Fop*-12	Utlou Lairem Leikai	24° 43.820’	93° 51.613’	2,517
13	*Fop*-13	Maibam Chingmang	24° 42.599’	93° 49.378’	2,525

### Morphological and molecular characterization of the isolates

### Radial growth, sporulation characterization of *Fop* isolates

**Figure 1:**
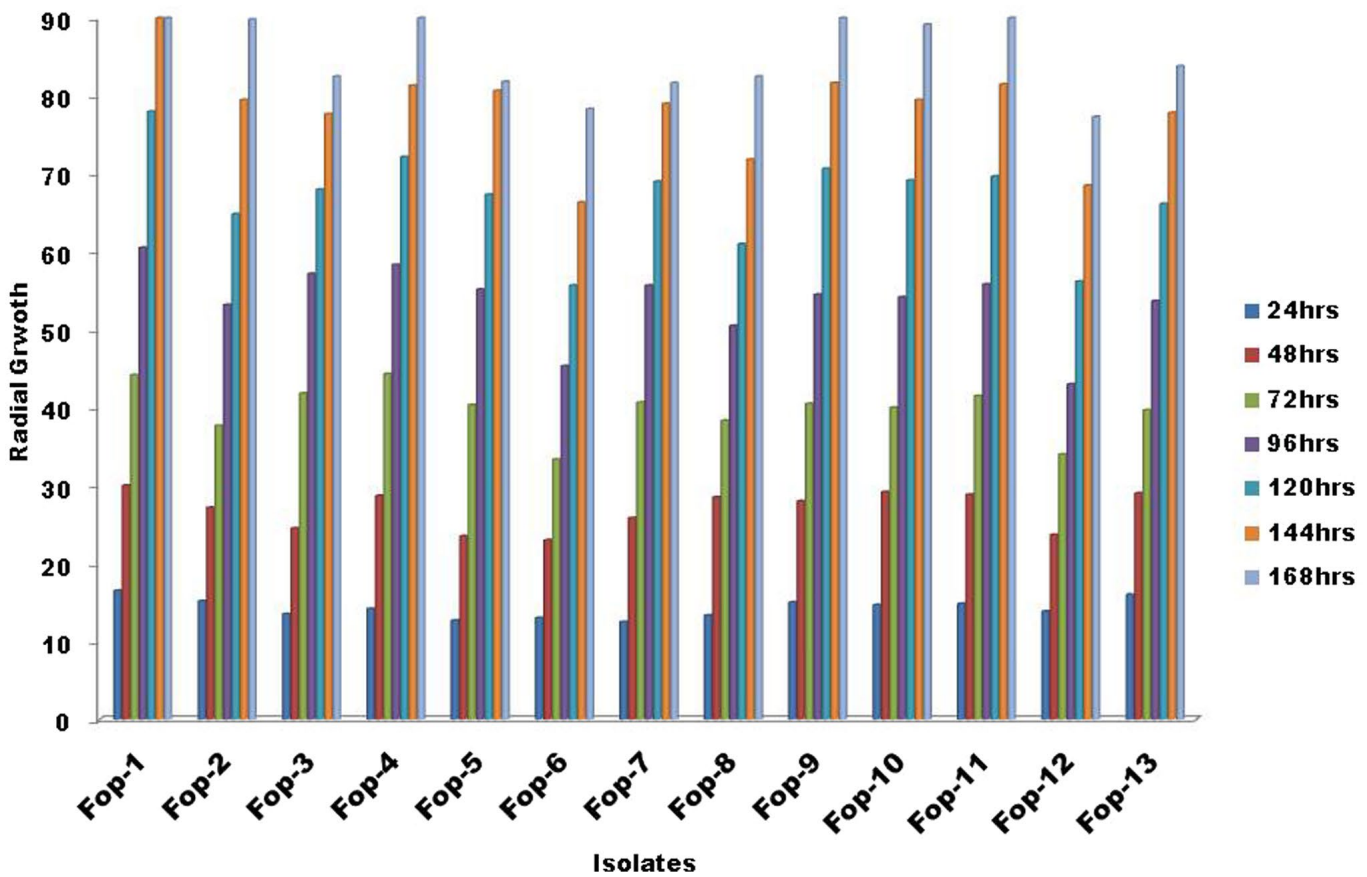
Radial growth of *Fop* isolates at specific intervals of time.

Estimation of radial growth of the isolates was performed on PDA plates, maximum radial growth was record isolate *Fop*-1 being 16.50 mm (24 h inoculation), 30.00 mm (48 h inoculation), 44.17 mm (72 h inoculation) was found (inoculation), 60.50 mm (96 h inoculation) and 78.00 mm (120 h inoculation). After 144 h of inoculation, it covered the entire plate (90.00 mm), considering it a fast growing isolate compared to the other isolates. Isolate *Fop*-6 recorded the smallest growth with 33.33 mm (72 h inoculation), 45.33 mm (96 h inoculation), 55.67 mm (120 h inoculation), 66.33 mm (144 h inoculation) and 78.33 mm (144 h vaccination). Significant variation between isolates was observed at certain time intervals. The growth rate of all isolates is shown in Figure 1. The *Fop* isolates showed huge variation in mycelial color. The color pattern ranged from off-white, matte white, and white gradually changing with different pigments, such as white to light pink, white to light purple, light pink to dark pink, purple with white paint, and light brown. The topography of the isolates ranged from thick to thin with less or slightly cottony mycelial growth as shown in Table 2. The pigmentation on the back ranged from light purple to dark purple, from light pink to deep pink and red. The marginal mycelial growth pattern ranged from regular to irregular. Formation of zones and the texture of the isolates are also shown in Table 2.

**Table 2:** Cultural characters of *Fop* isolates.

Isolates	Mycelial color	Topography	Pigmentation (Rear side)	Type of margin	Concentric Zones	Texture
*Fop*-1	Light purple with white color	Thick mycelium with cottony growth	Light purple	Regular	Present	Fluffy
*Fop*-2	Light pink	Thin mycelium with cottony growth	Dark pink	Regular	Absent	Slightly fluffy
*Fop*-3	Light purple with white color	Thin mycelium with less cottony growth	Dark purple	Irregular	Absent	Fluffy
*Fop*-4	White	Thick mycelium with cottony growth	Dark pink	Regular	Absent	Fluffy
*Fop*-5	White	Thick mycelium with cottony growth	Light purple	Regular	Absent	Fluffy
*Fop*-6	Purple with white color	Thick mycelium with slightly cottony growth	Dark purple	Irregular	Absent	Slightly fluffy
*Fop*-7	Off white	Thin mycelium with less cottony growth	Deep red	Regular	Present	Fluffy
*Fop*-8	Light purple with white color	Thick mycelium with less cottony growth	Dark purple	Irregular	Absent	Fluffy
*Fop*-9	Pink and white color	Thick mycelium with cottony growth	Purple	Regular	Present	Fluffy
*Fop*-10	Dull white	Thin mycelium with cottony growth	Light pink	Regular	Absent	Fluffy
*Fop*-11	The white color in the middle and purple at edges	Thick mycelium with less cottony growth	Dark purple	Irregular	Absent	Fluffy
*Fop*-12	Light pink with light red color	Thick mycelium with cottony growth	Deep red	Regular	Absent	Fluffy
*Fop*-13	Light pink	Thin mycelium with less cottony growth	Dark purple	Regular	Present	Slightly fluffy

**PLATE 1:**
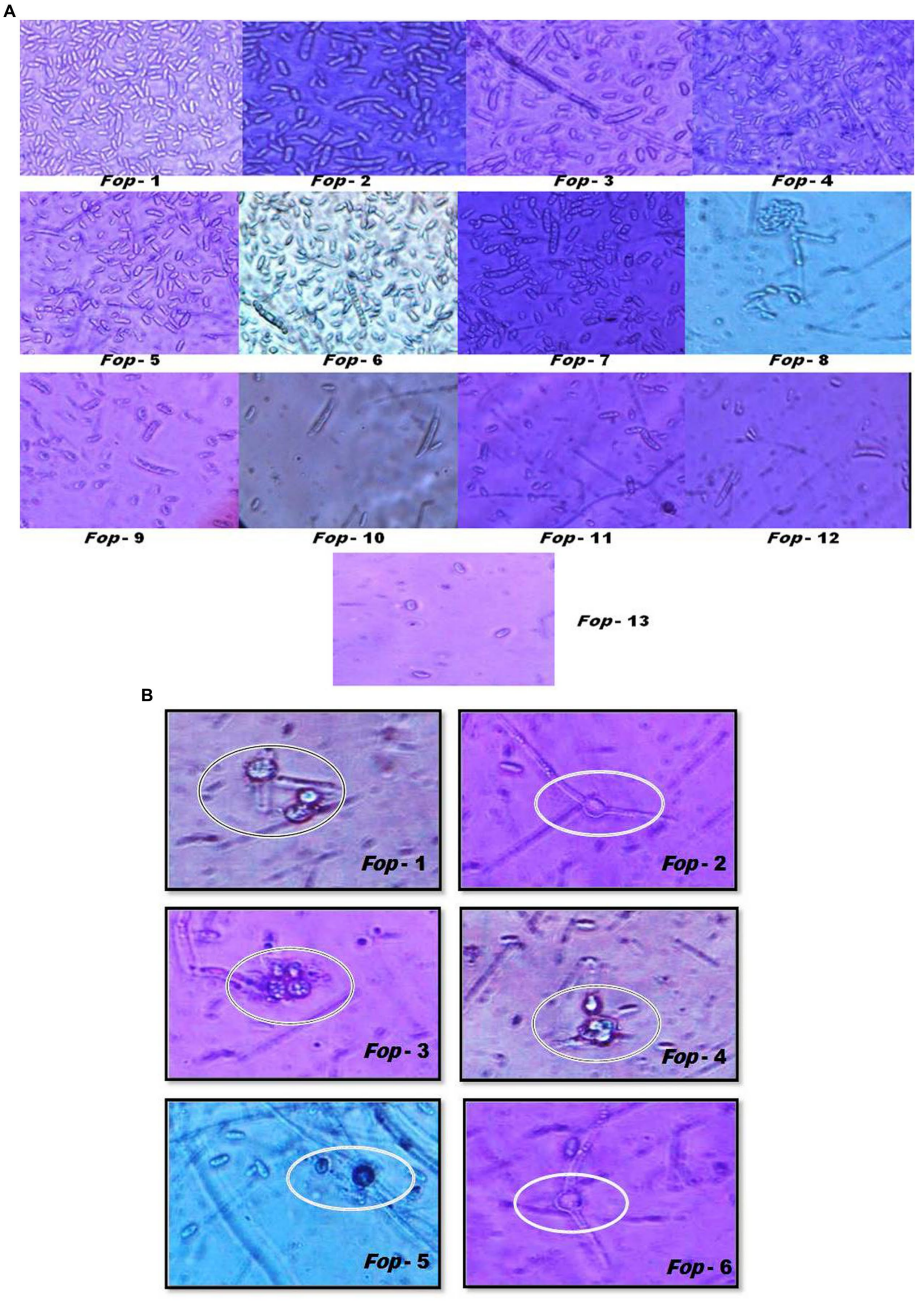
**(A)** Sporulation (Microconidia and Macroconidia) and their characterization of different *Fop* isolates (40X magnification). **(B)** Sporulation (Chamydospores) and their characterization of different *Fop* isolates (40X magnification).

*Fop* isolates can typically be characterized by their spores (Plates 1a,1b). *Fop* isolates tend to form micro, macro and chlamydospores. The characterization of *Fop* isolates for conidia is presented in Table 3. It lists the spore count, septum count, size and shape. All isolates showed obvious differences in spore number, septum color, size and shape.

**Table 3:** Sporulation and characterization of *Fop* isolates.

Isolates	Macro conidia*				Micro conidia*				Chlamydospore*		
	Size LxB (μm)	Septation	Sporulation/mL (×10^5^)	Shape	Size LxB (μm)	Septation	Sporulation/mL (×10^5^)	Shape	Daimeter (μm)	Formation	Shape
*Fop*-1	13.53 × 3.41	3–4	39.5	Sickle-shaped with pointed ends	8.45 × 3.93	0	6.80	Oval to elliptical	5.37	Intercalary	Globose
*Fop*-2	14.70 × 3.51	2–3	26.40	Sickle-shaped with blunt ends	4.73 × 2.30	0	1.10	Elliptical	6.33	Terminal	Globose
*Fop*-3	19.22 × 4.33	3–4	38.7	Sickle-shaped with blunt ends	7.94 × 3.85	0	3.90	Round to oval, oval to elliptical	5.64	Terminal	Globose
*Fop*-4	19.31 × 3.25	3–4	15.40	Sickle-shaped with blunt ends	7.93 × 2.28	0–1	2.10	Elliptical	7.21	Terminal	Globose
*Fop*-5	14.72 × 3.98	3–4	26.6	Slightly curved with blunt ends	8.15 × 3.87	0	4.50	Round to oval, oval to elliptical	7.65	Intercalary	Globose
*Fop*-6	21.23×3.89	2–3	30.50	Short, straight with blunt ends	9.48 × 3.75	0	5.40	Round to oval, oval to elliptical	6.15	Terminal and intercalary	Globose
*Fop*-7	11.95 × 4.98	2–3	16.0	Short, straight with blunt ends	9.64 × 3.62	0	1.40	Oval to elliptical	5.31	Terminal	Globose
*Fop*-8	16.34 × 5.21	1–3	22.80	Short, straight with blunt ends	8.90 × 4.72	0	2.80	Round to oval, oval to elliptical	4.83	Terminal	Globose
*Fop*-9	12.48 × 3.75	1–2	13.90	Short, straight with blunt ends	7.53 × 3.82	0	1.20	Round to oval	5.60	Intercalary	Globose
*Fop*-10	18.25 × 4.52	3–4	21.50	Sickle-shaped with pointed ends	9.23 × 2.79	0–1	1.90	Elliptical	5.23	Intercalary	Globose
*Fop*-11	15.86 × 3.85	3	20.50	Short, straight with blunt ends	8.62 × 4.10	0	2.40	Elliptical	5.48	Terminal	Globose
*Fop*-12	21.24 × 3.54	3–4	23.90	Slightly curved with blunt ends	8.24 × 4.31	0	4.50	Oval	7.43	Intercalary	Globose
*Fop*-13	24.24 × 3.96	2–4	15.00	Sickle-shaped with blunt ends	8.78 × 4.92	0–1	1.40	Oval	4.89	Terminal and intercalary	Globose

* Mean of 10 replications.

L, length, B, breadth.

### Molecular characterization of *Fop* isolates

**PLATE 2:**
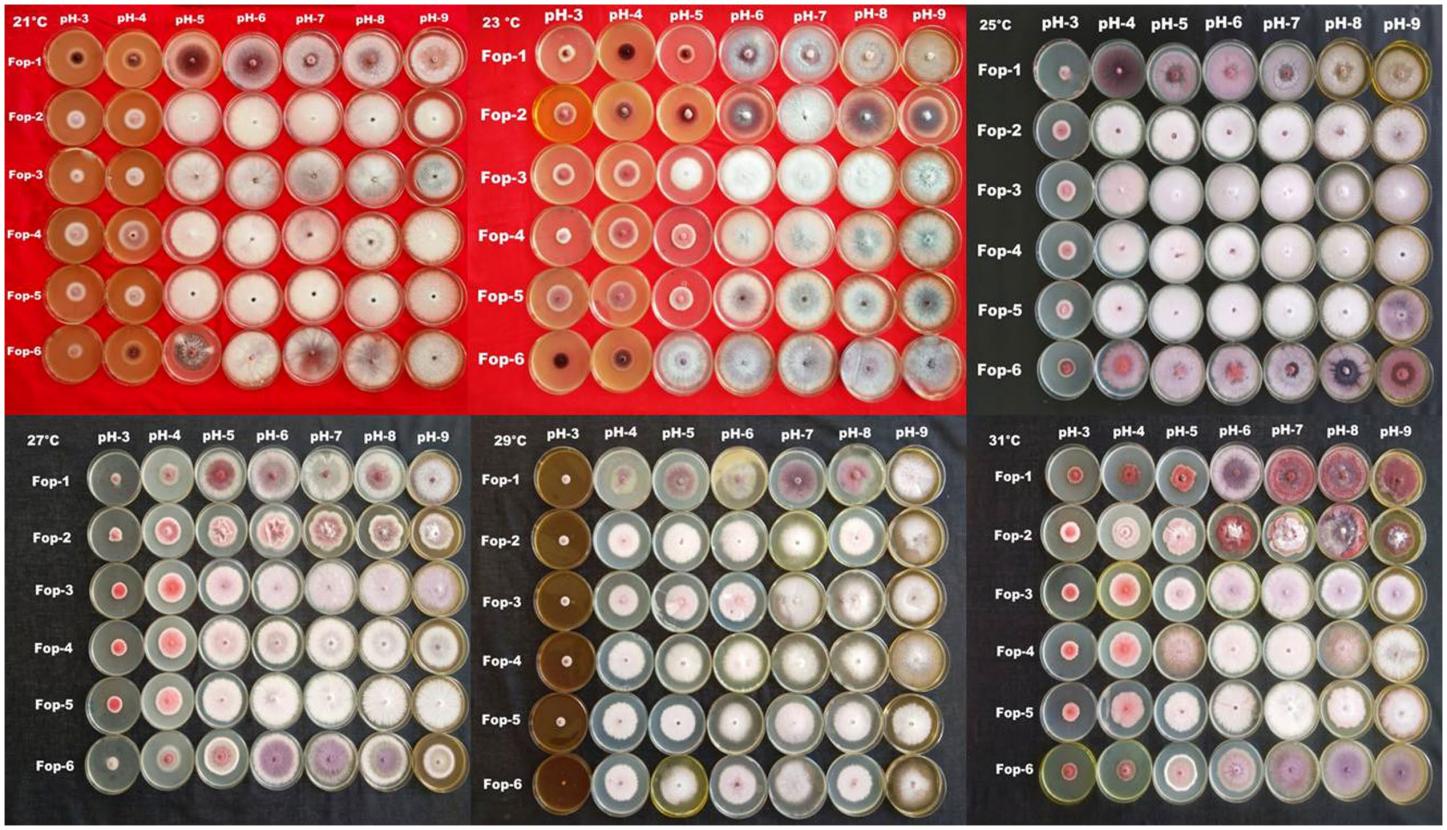
PCR amplification of ITS region of *Fop* isolates (*L* = 100 bp Ladder, 1–13 = *Fop* isolates).

**Figure 2:**
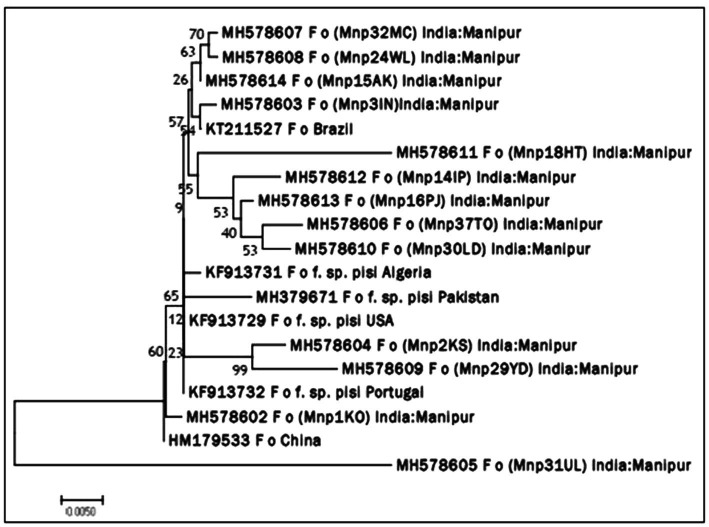
Neighbor joining (NJ) phylogenetic tree illustrating the concatenated (16S rDNA) sequence based phylogenetic relationship of *Fop* isolates.

**PLATE 3:**
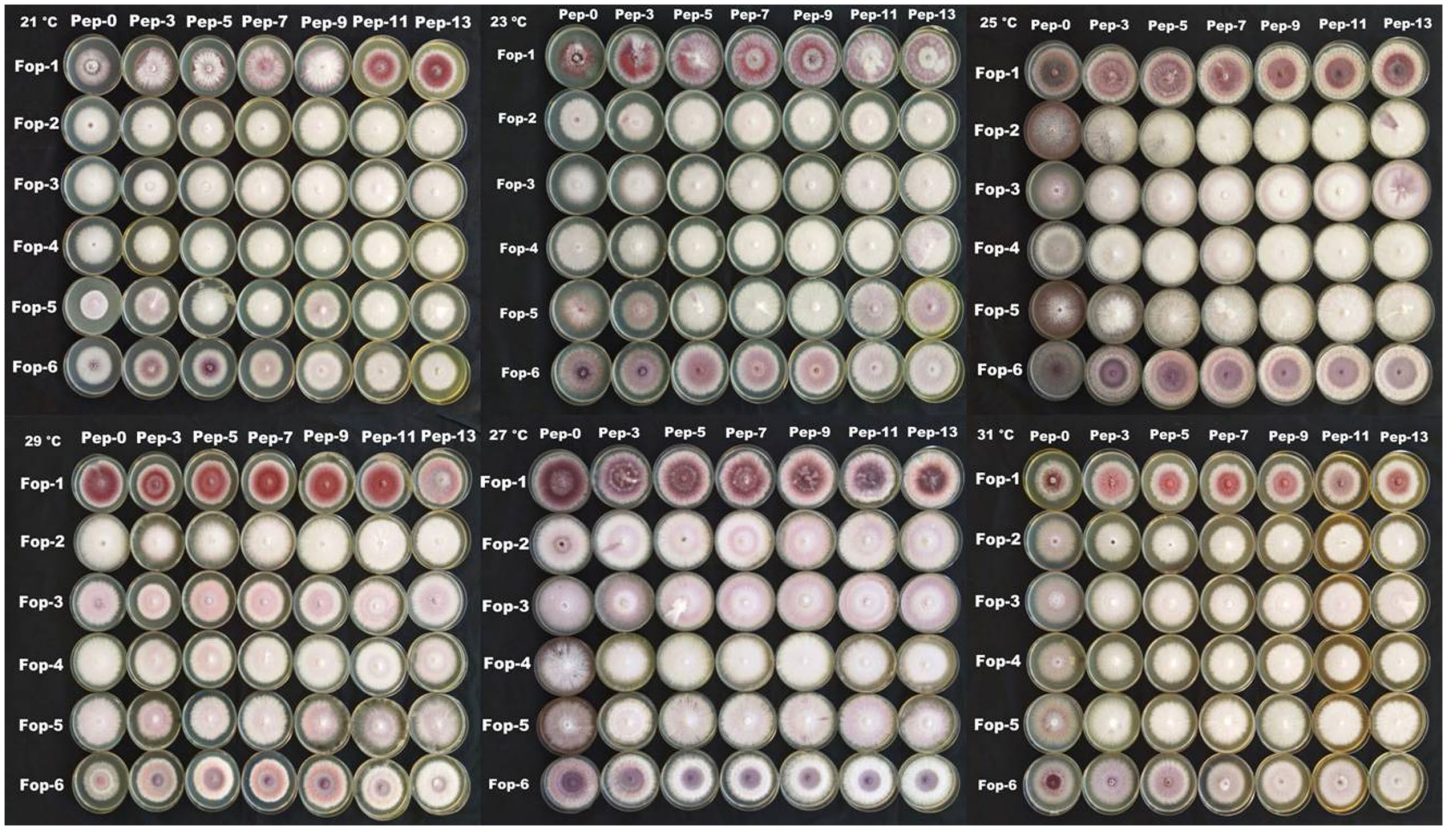
Combination effect of temperature and pH on *Fop* isolates.

**PLATE 4:**
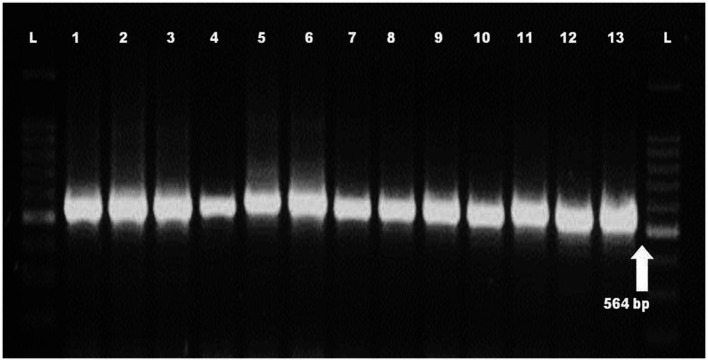
Combination effect of temperature and Nitrogen levels on *Fop* isolates.

Molecular identification of *Fop* isolates was performed by extraction of total genomic DNA, PCR amplification using the universal primers ITS1 and ITS4 primers. Approximately 564 nucleotides were amplified by PCR and confirmed by 1.2% agarose gel electrophoresis (Plate 2). The ITS region sequence of 13 isolates of *Fusarium* species has been submitted to the NCBI and the isolates have been assigned accession numbers, namely (MH578602), (MH578602), (MH578603), (MH578604), (MH578605), (MH578606), (MH578607), (MH578608), (MH578609), (MH578610), (MH578611), (MH578612), (MH578613) and (MH578614). The ITS sequence of the isolates was searched for their homologous sequences in publicly available databases and analyzed with the nucleotide BLAST software. Molecular Evolutionary Genetics Analysis (MEGA 7) software was used for phylogenetic analysis. The obtained nucleotide sequences were assembled and aligned using the ClustalW integrated in MEGA 7, and the phylogenetic tree was constructed using the neighbor-joining method. The evolutionary history was inferred using the neighbor-joining method performed in MEGA7 (Figure 2). The bootstrap consensus tree derived from 1,000 replicates was used to represent the evolutionary history of the *Fop* isolates from Manipur. Analysis included 19 nucleotide sequences using 13 isolates of *F. oxysporum* from Manipur and 6 isolates from a gene bank from Brazil, Algeria, Pakistan, the United States, Portugal and China and other parts of the world. The tree has two separate groups, one consisting of isolate Mnp31UL and the rest of the isolates were found in another group, showing that isolate Mnp31UL was the most diverse among all isolates compared. In the present study, isolates Mnp2KS and Mnp29YD, isolates Mnp37TO and Mnp30LD, and isolates Mnp32MC and Mnp24WL were each found to belong to one group, showing a highly related ancestry (Plates 3, 4).

### Optimization studies to evaluate the growth of different *Fop* isolates at different temperatures and different pH levels

**Figure 3:**
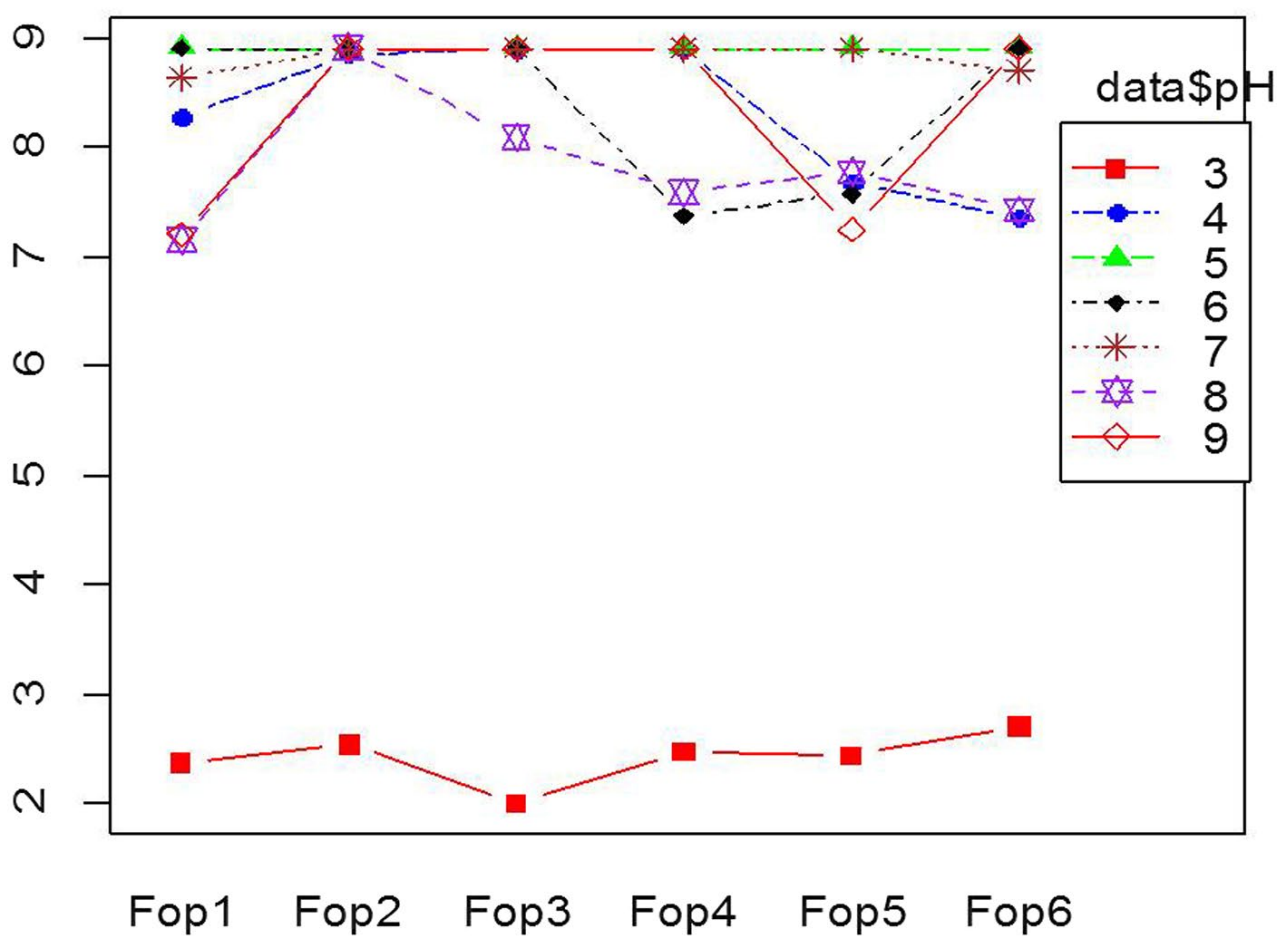
Interaction plot of radial growth of six *Fop* isolates and their growth at various levels of pH.

The test to achieve optimized pH conditions for the growth of *Fop* isolates gave significant results. *Fop* isolates were grown on PDA medium whose pH was changed within the pH range tested against the growth of six *Fop* isolates; pH5 gave the best growth results when the radial growth of all isolates after 7 days of inoculation was a maximum of 9 cm and covered the entire plate. The smallest radial growth of all isolates was recorded at pH3. Radial growth of all isolates decreased and was limited to 2.5–3 cm in isolates, making a pH of 3 unsuitable. pH values of 4, 6, 7, 8, 9 showed growth of 7–9 cm, which varied between the isolates. The different growth of all *Fop* isolates with different pH values is shown in the interaction diagram Figure 3. The results obtained were consistent with the researchers findings (Attri et al., 2018; David et al., 2019; Somesh et al., 2019; Chaithra et al., 2020). The results obtained are supported by the results of Groenewald et al. (2006) who carried out *in vitro* studies on the radial growth of other *Fusarium* species and *Formae* special species. *Fusarium oxysporum* sp. *cubense* showed maximum radial growth at pH6 and 25°C, with minimal growth at pH4 using the same citrate/phosphate buffer medium.

**Figure 4:**
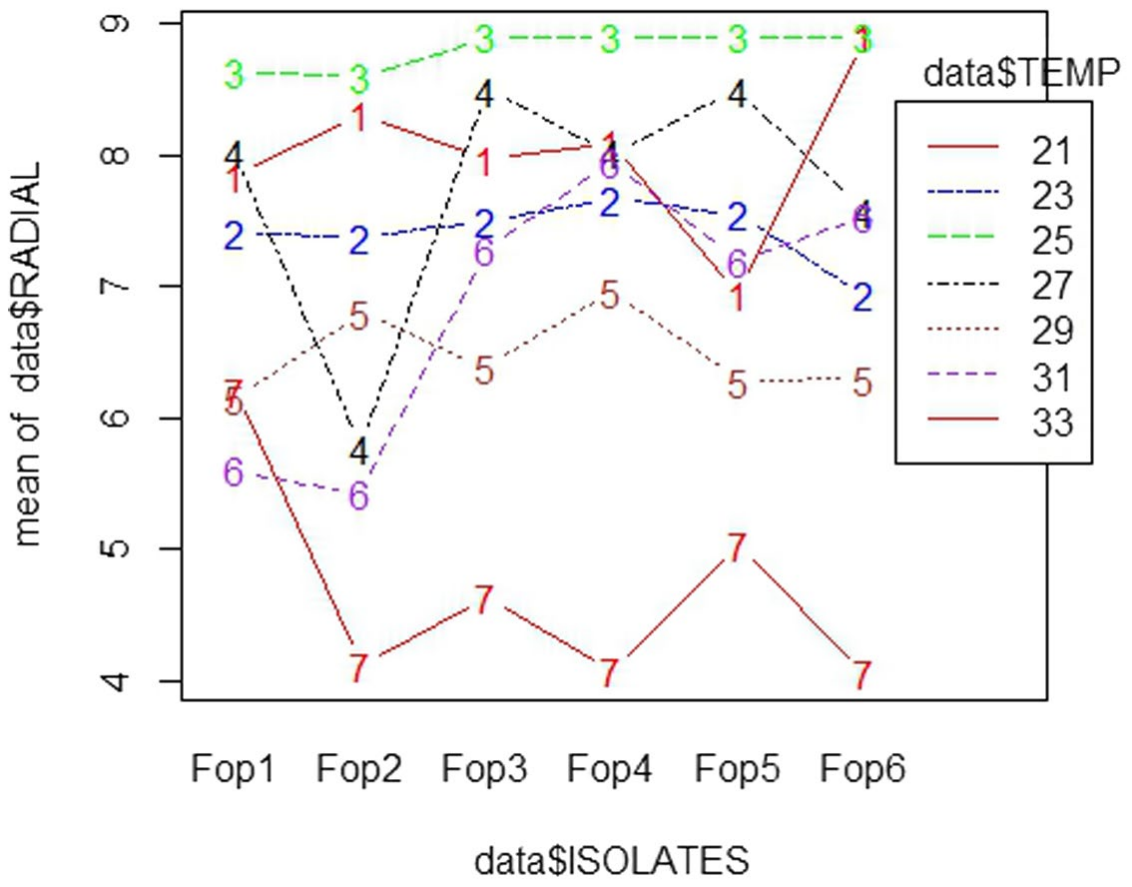
Interaction plot of radial growth of six *Fop* isolates and their growth at various levels of Temperatures.

Among all temperatures examined against the radial growth, a temperature of 25°C proved good and supported the growth of *Fop* isolates, with maximum growth being 8.8–9 cm. The lowest growth of all isolates was observed at 33°C, with the growth of all isolates dropping to 4 cm among isolates. The interaction diagram revealed that the growth of each isolate varied at different temperatures. The growth of *Fop* isolates at different temperatures is shown in the interaction diagram with different isolates and different temperatures, Figure 4. The influence of temperature on the *Fop* results has been compared with the results of David et al. (2019), Somesh et al. (2019), and Attri et al., 2018. Similar results for optimal fungal growth at a temperature of 25°C were reported for *Fusarium oxysporum* sp. *lactucae*, *Fusarium oxysporum* sp*. fabae* and *Fusarium oxysporum* sp. *spinaciae* (Naiki and Morita, 1983; Ivanovic et al., 1987; Scott et al., 2010).

### Combination effect of temperature and pH, temperature and nitrogen dose levels on radial growth on six isolates of *Fop*

**Figure 5:**
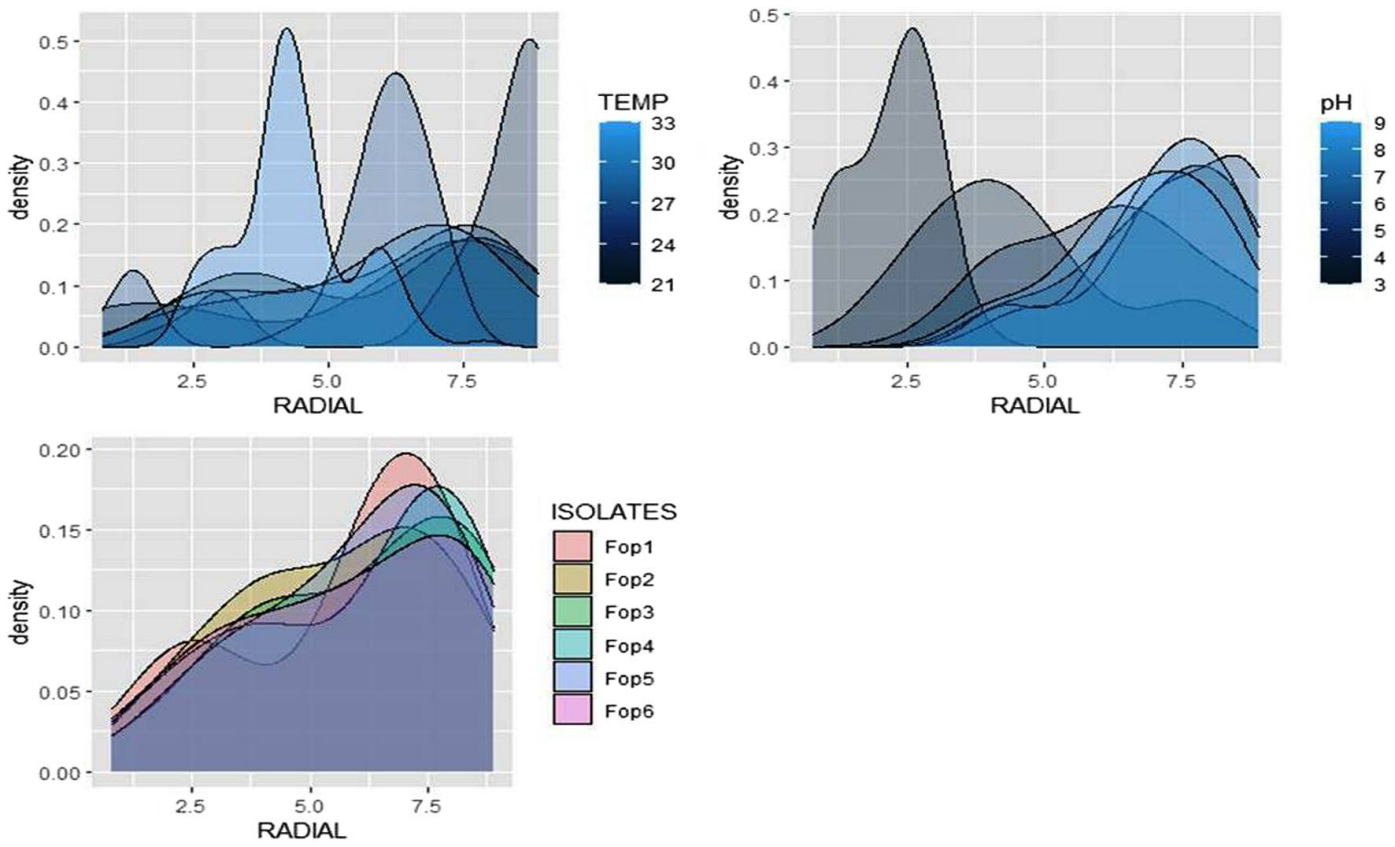
Density graph of radial growth of six *Fop* isolates with synergistic effect of Temperature and pH.

The radial growth of *Fop* isolates resulted in a variable trend in response to combined changes in temperature and pH. All isolates showed increasing radial growth patterns with increasing temperature and pH. The increasing pattern was observed in all *Fop* isolates at a temperature of 21–27°C with a change in pH from 4 to 8. The mean radial growth of all isolates in relation to different temperatures ranged from 5.76 to 6.13 cm, with isolate *Fop*-3 having the highest proportion, followed by *Fop*-4, *Fop*-5, *Fop*-6, *Fop*-1, and *Fop*-2. The radial growth of the isolates at different temperatures ranged from 4.35 to 7.66 cm, with the highest mean radial growth of 7.66 cm of all isolates observed at 25°C. Isolate *Fop*-3 showed the strongest radial growth of all isolates in relation to different pH values and the smallest radial growth in *Fop*-1. The mean radial growth at different pH values for isolates, the strongest radial growth was observed at pH7. The growth of all isolates with regard to temperature and pH should be at a maximum at the combination of 25°C and pH7, respectively. Different importance of mean radial growth among isolates was observed. A wide variation in the radial growth of all isolates with changes in temperature and pH was observed. The density plot of the isolates at different temperatures and pH showed differences between isolates, temperature and pH. Growth densities of all isolates were concentrated at 5.5 to 7 cm. The density plot (Figure 5) also shows the variation in radial growth between isolates as the growth of the isolates is not equal. The results obtained are supported by David et al. (2019). The variation exhibited by the isolates at different temperatures and pH values was not limited to radial growth, but there were variations between isolates in terms of fungal growth pattern, color changes/pigmentation of the mycelium and substrate. Its changes can be visualized in plate: 3.

**Figure 6:**
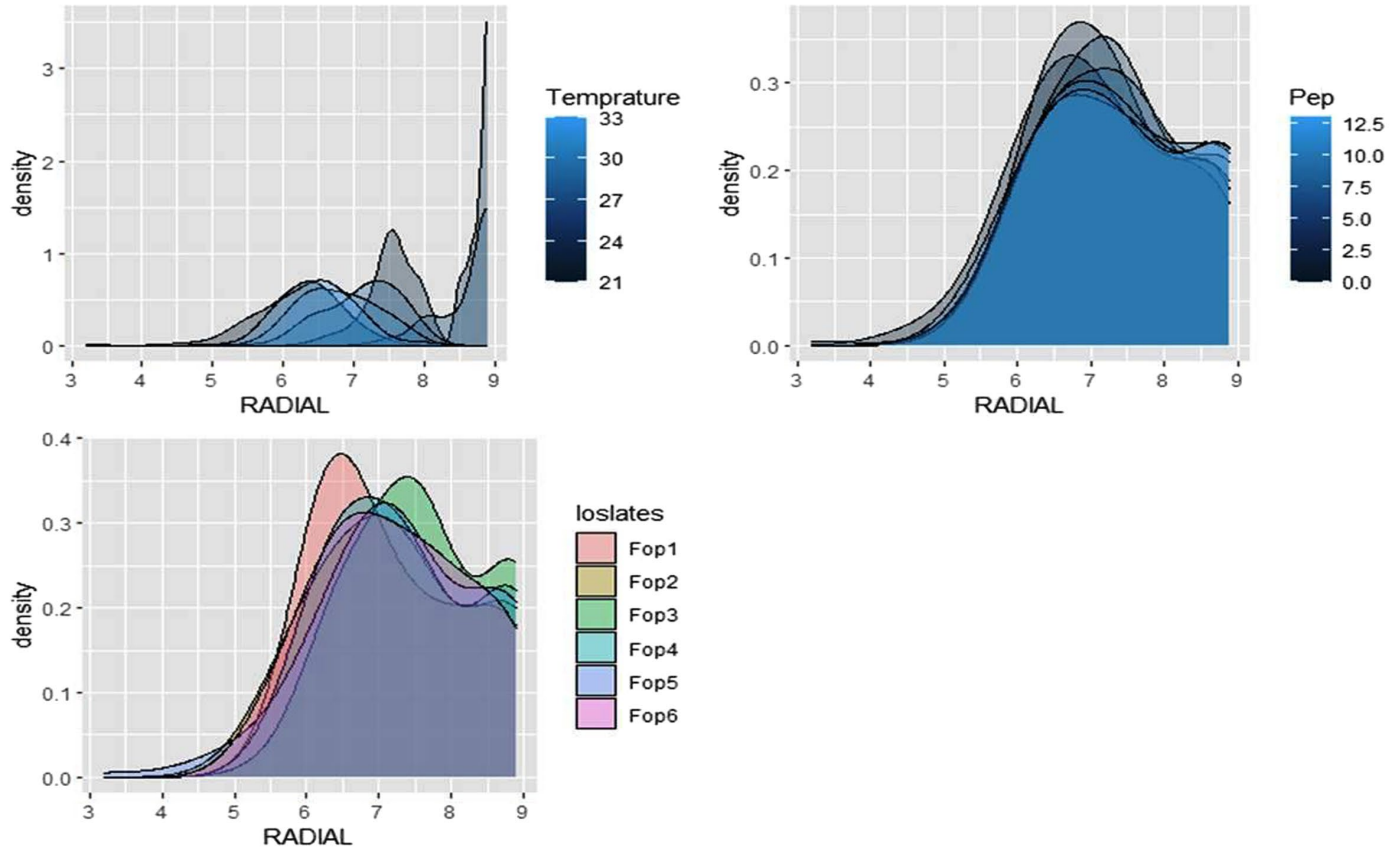
Density graph of radial growth of six *Fop* isolates with synergistic effect of Temperature and Nitrogen dose.

The radial growth of *Fop* isolates resulted in a variable trend in response to combined changes in temperature and nitrogen doses. All isolates showed increasing radial growth patterns with increasing temperature and nitrogen dose. The increasing pattern was observed in all *Fop* isolates at a temperature of 21–27°C with a change in nitrogen dose. The maximum mean radial growth (8.9 cm) of all isolates was observed at a temperature of 25°C and 27°C with nitrogen doses of 9, 11, and 13 g, respectively. The mean radial growth of all isolates with respect to temperature was 7.62 cm for isolate *Fop*-3, followed by *Fop*-4, *Fop*-5, *Fop*-2, and *Fop*-1. Maximum growth at different temperatures for isolates was observed at 25°C. The highest mean radial growth of the isolates for changing media supplementation with different nitrogen dose levels, the highest radial growth showed isolate *Fop*-3 and the lowest isolate *Fop*-1. The radial growth of *Fop* isolates was found to be maximal at a temperature and nitrogen dose of 25°C and 11 g. Difference in mean radial growth among isolates was observed. A large variation in the radial growth of all isolates with changes in temperature and nitrogen dose was also observed. The density diagram of the isolates at different temperatures and different nitrogen doses showed differences between the isolates, the temperature and the pH. Growth densities of all isolates were concentrated at 5 to 8 cm. The density plot (Figure 6) also shows the variation in radial growth between isolates as the growth of the isolates is not equal. The results obtained are supported by David et al. (2019). Notably, the variation was observed not only in radial growth but also in patterns of mycelial growth and pigmentation. Its changes can be visualized in plate: 4.

## Discussions

The study provides evidence that all *Fop* isolates collected and isolated in different valley districts of Manipur showed mutual differences in morphology such as growth, mycelial properties, sporulation, size and shape. Colony character such as mycelial color (white, light pink, light purple, light brown), topography (thin, thick, cottony or non-cottony), texture (slightly fluffy to fluffy), border and pigmentation produced were recorded and all isolates detected showed large differences between them. The differences in cultural traits observed in *Fop* cultures have been important for the biology of fungi as they occur in nature because it is closely linked to the question of the physiological races of the pathogens. The results obtained in our investigation were consistent with the previous results of many workers (Singh et al., 2010; Anser and Srivastava, 2013; Ashwathi et al., 2017; Thaware et al., 2017). Differences in sporulation, size, shape, color and number of septations of macroconidia and microconidia, and chlamydospore diameter were observed among Fusarium isolates. These observations indicate that sporulation is important for the virulence of the isolates. The isolates that produced heavy sporulation were highly virulent, while isolates that produced low to moderate sporulation had very low pathogenicity. All morphological characters of *Fusarium oxysporum* obtained in the present investigation agree broadly with Booth (1971) and Nelson et al. (1990). A maximum degree of variability has previously been reported by former investigators in *Fusarium oxysporum* sp. *pisi* (Linford, 1928; Kraft, 1994). Awachar (2014) and Thaware et al. (2017) also described the size of microconidia, macroconidia and chlamydospores of Fusarium wilt of chickpeas. The current results confirm the reports of previous investigators who based their morphological studies on appropriate media for the growth and sporulation of Fusarium spp. (Kulkarni, 2006).

The characterization of the population structure of fungal pathogens is important for understanding the biology of the organism and for the development of disease control strategies (Malvick and Percich, 1998), as well as for molecular studies of individuals, which are one of the components of the population structure (Leung et al., 1993). However, the classification system based solely on morphology does not provide an accurate tool for identifying species. Therefore, a molecular approach is one of the most promising detection methods for species identification. In the present study, the morphological characterization of the isolates belonged to Fusarium spp. and their identity was confirmed using molecular methods. The primer combination ITS1 and ITS4 generated a fixed region length of 564 bp for 13 *Fusarium oxysporum* isolates. By comparing their sequences to the NCBI database using a blast search, a complete query with the highest match and coverage for *Fusarium oxysporum* was found. Chanu et al. (2018) worked on the molecular identification of *Fusarium oxysporum* f.sp. ciceris from chickpeas and confirmed that using the primer combination ITS1 and ITS4 resulted in a fixed region length of 564 bp. Devi et al. (2016) reported that the ITS region was successfully amplified from DNA of *Fusarium oxysporum* strains. Joshi et al. (2013) confirmed 39 isolates of *Fusarium oxysporum* using species-specific primers FOF1 and FOR1, which amplified the 340 bp DNA fragment. The Internal Transcribed Spacer (ITS) region is possibly the most frequently sequenced DNA region in fungi and is phylogenetically interpretable, i.e., the sequences are comparatively easy to align and large enough to provide potentially useful features for phylogenetic reconstruction. PCR amplification of the ITS region has become a popular choice for phylogenetic analysis of closely related species and populations. ITS1 and ITS4 of ribosomal RNA, which encodes DNA, have numerous advantages that represent an ideal region for a sequence for phylogenetic analysis. Its speed of development lends itself to study at both the specific and general levels. Therefore, the PCR-ITS method described in this study provides a simple and rapid procedure to differentiate *Fusarium* strains at the species level.

Optimization studies on six *Fop* isolates revealed that temperature and pH are key factors determining fungal growth. All *Fop* isolates were shown to show maximum growth at a temperature of 25°C and a neutral and slightly acidic medium. It has been observed that optimal temperatures and pH support the growth of the fungus for it to thrive and that the fission changes in the factors can alter the growth of the fungus. This was observed when lower and higher temperatures and higher pH were used. A reduction in the growth of the fungus with changes in its growth pattern and morphology was observed. These results also show that isolates found in the warmer regions tend to survive harsh conditions compared to isolates that thrive in temperate climates. The soils of Northeast India exhibit acidic conditions that favor the growth of the *Fop*. This in turn helps the fungus survive longer in the form of dormant structures. Our results on the influence of temperature and pH on fungal growth were consistent with the findings of several investigators studying Fusarium spp. All results indicated that the optimal temperature and pH for fungal growth were 25–30°C and 4–7°C, respectively (Groenewald et al., 2006; Wu et al., 2009; Gupta et al., 2010). However, temperature and pH may not be the only factors influencing the growth of the fungus. Other factors such as relative humidity and water potential may also have some impact on growth promotion (Cochrane, 1958; Nelson et al., 1990). The strongest radial growth tends to occur at 25°C. in *Fusarium oxysporum* sp. Betae isolates collected from temperate regions in the United States (Webb et al., 2015) and for *Fusarium oxysporum* sp. Psidii and F. solani isolates collected from subtropical regions in India (Gupta et al., 2010).

The merging effect of temperature, pH, temperature and nitrogen content shows that *Fop* isolates collected from different sites in the valley districts of Manipur tend to have different growth rates under different conditions of temperature, pH and nitrogen content base to achieve. Total *Fop* isolates were observed to exhibit white, cottony, fluffy mycelial growth as the nitrogen content of the basal medium increased, suggesting that field nitrogen concentrations support vegetative growth of the disease-causing pathogens. It was observed that the fungal isolates changed their morphology with changes in temperature, pH and nitrogen content (Panels 3 and 4). From this it can be understood that the change in climatic conditions causes mutations or genetic changes in pathogenic fungi. The climate-related changes of the fungus may pose a serious threat to the living world. As pathogens become more virulent under the influence of these changing climate scenarios, new epidemics can arise that can starve people and animals and cause devastating problems in the human world. The higher temperatures and pH levels and indiscriminate fertilization in the fields may not have an immediate impact but may pose future challenges that may be difficult to manage.

## Future aspects

Since the results of inter-isolate variation with changes in temperature, pH and nitrogen doses are confirmed at the morphological level, these should also be evaluated at the genetic level to determine the degree of inter-isolate variation when there is a change in atmospheric conditions. The same should be evaluated under field conditions involving the host to determine the effect of the pathogenic isolates.

## Data availability statement

The original contributions presented in the study are included in the article/supplementary material, further inquiries can be directed to the corresponding author.

## Author contributions

All authors listed have made a substantial, direct, and intellectual contribution to the work and approved it for publication.

## Funding

The authors would like to express their sincere gratitude to the College of Agriculture, Central Agricultural University, Imphal, India for financial support to keep the research running smoothly.

## Conflict of interest

The authors declare that the research was conducted in the absence of any commercial or financial relationships that could be construed as a potential conflict of interest.

## Publisher’s note

All claims expressed in this article are solely those of the authors and do not necessarily represent those of their affiliated organizations, or those of the publisher, the editors and the reviewers. Any product that may be evaluated in this article, or claim that may be made by its manufacturer, is not guaranteed or endorsed by the publisher.

## References

[ref1] Anonymous. (2019). Climate change and agriculture: a perfect storm in farm country. Union of concerned scientists. Available at: https://www.ucsusa.org/resources/climate-change-and-agriculture (Accessed March 20th, 2019).

[ref2] Anser M. Srivastava M. (2013). Morphological variability and pathogenic reactions of *fusarium oxysporum* f.sp. *ciceri* isolates to cultivars of chickpea. Ann. Pl. Prot. Sci. 21, 345–348.

[ref06] Armstrong G. M. Armstrong J. K. (1974). Races of *Fusarium oxysporum* f.sp. pisi causal agents of wilt of pea. Phytopathol 64, 849–857.

[ref3] Ashwathi S. Ushamalini C. Parthasarathy S. Nakkeeran S. (2017). Morphological and molecular characterization of *fusarium* spp. associated with vascular wilt of coriander in India. J. Pharmaco. Phytochem 6, 1055–1059.

[ref4] Aslam S. Ghazanfar M. U. Munir N. Hamid M. I. (2019). Managing fusarium wilt of pea by utilizing different application methods of fungicides. Pakistan J. Phytopathol. 31, 81–88. doi: 10.33866/phytopathol.031.01.0482

[ref5] Attri K. Sharma M. Gupta S. K. (2018). Influence of edaphic factors on fusarium wilt of bell pepper. Int. J. Bio-Reso. 9, 606–610. doi: 10.23910/IJBSM/2018.9.5.1904

[ref6] Awachar M.K. (2014). Studies on morphological variability of *fusarium oxysporum* f.sp. *ciceris* causing wilt of chickpea. M.Sc. (Agri.) thesis, MPKV, Rahuri (India).

[ref7] Bani M. Cimmino A. Evidente A. Rubiales D. Rispail N. (2018). Pisatin involvement in the variation of inhibition of *fusarium oxysporum* f. sp. *pisi* spore germination by root exudates of *Pisum* spp germplasm. Pl. Pathol. 67, 1046–1054. doi: 10.1111/ppa.12813

[ref01] Booth C. (1971). The genus *Fusarium*. Kew, Surrey, England: Common Wealth Mycological Institute, 114.

[ref9] Brake V. M. Pegg K. G. Irwin J. A. G. Chaseling J. (1995). The influence of temperature, inoculum level and race of *fusarium oxysporum* f.sp. *cubense* on the disease reaction of banana cv Cavendish. Aust. J. Agric. Res. 46, 673–685. doi: 10.1071/AR9950673

[ref02] Buxton E. W. Storey I. F. (1954). The occurrence of pea wilt in Britain. Pl. Pathol. 3, 13–16.

[ref10] Chaithra J. Kulkarni S. Sunkad S. B. Amresh Y. S. Shekhar P. (2020). Effect of different media, temperature, pH and illuminaton on the growth *of fusarium oxysporum* f. sp. *zingiberi* causing wet rot of ginger. J. Pharmacog. Phytochem. 9, 786–790.

[ref11] Chanu W. T. Sinha B. Devi P. S. Devi N. O. Devi H. C. (2018). Molecular identification of pathogens associated with soil borne diseases of chickpea and their management by native *Trichoderma* spp. Pl. Dis. Res. 33, 50–59.

[ref12] Chen L. H. Huang X. Q. Yang X. M. Shen Q. R. (2013). Modeling the effects of environmental factors on the population of *fusarium oxysporum* in cucumber continuously cropped soil. Commun. Soil Sci. Plant Anal. 44, 2219–2232. doi: 10.1080/00103624.2012.760577

[ref13] Chupps C. Sherf A.F. (1960). Vegetable disease and their control. The Ronald Press Company, New York, pp. 668.

[ref14] Cochrane V. (1958). “Cultivation and growth” in Physiology of fungi (New York, NY: Wiley), 1–27.

[ref15] David R. Cruz L. L. F. S. Munkvold G. P. (2019). Effects of Temperature and pH on *Fusarium oxysporum* and Soybean Seedling Disease. Pl. Dis. 103, 3234–3243. 10.1094/PDIS-11-18-1952-RE 31573433

[ref16] Deng D. Sun S. Wu W. Zong X. Yang X. Zhang X.. (2022). Screening for pea germplasms resistant to Fusariu wilt race 5. Agron. 12:1354. doi: 10.3390/agronomy12061354

[ref17] Devi D. N. Venkataramann M. Srivastava R. K. Uppalapati S. R. Gupta V. K. YliMuttila T.. (2016). Molecular phylogeny, pathogenicity and toxigenicity of *fusarium oxysporum* f.sp. *lycopersici*. Sci. Rep. 6:21367. 26883288 10.1038/srep21367 PMC4756691

[ref18] Ekvall J. Stegmark R. Nyman M. (2006). Content of low molecular weight carbohydrates in vining peas (*Pisum sativum*) related to harvest time, size and brine grade. Food Chem. 94, 513–519. doi: 10.1016/j.foodchem.2004.11.044

[ref19] El-Sharkawy H. H. A. Abbas M. S. Soliman A. S. Ibrahim S. A. El-Nady I. A. I. (2021). Synergistic effect of growth-promoting microorganisms on bio-control of *fusarium oxysporum* f. sp. *pisi*, growth, yield, physiological and anatomical characteristics of pea plants. Pestic. Biochem. Physiol. 178:104939. doi: 10.1016/j.pestbp.2021.104939, PMID: 34446206

[ref05] FAO, Food and Agriculture Organization. (2020). Available at: https://www.fao.org/faostat/en/#data/QCL.

[ref20] Gatch E. W. du Toit L. J. (2017). Limestone-mediated suppression of fusarium wilt in spinach seed crops. Plant Dis. 101, 81–94. doi: 10.1094/PDIS-04-16-0423-RE, PMID: 30682316

[ref21] Groenewald S. Berg N. V. D. Marasas W. F. O. Viljoen A. (2006). Biological, physiological and pathogenic variation in a genetically homogenous population of *fusarium oxysporum* f.sp. *cubense* austral. Plant Pathol. 35, 401–409. doi: 10.1071/AP06041

[ref22] Gupta V. K. Misra A. K. Gaur R. K. (2010). Growth characteristics of *fusarium* spp. causing wilt disease in *Psidium guajava* L. in India. J. Plant Prot. Res. 50, 452–462.

[ref23] Haglund W. A. Kraft J. M. (1970). *Fusarium oxysporum* f. *pisi* race 5. Phytopathology 60, 1861–1862. doi: 10.1094/Phyto-60-1861

[ref24] Haglund W. A. Kraft J. M. (1979). *Fusarium-oxysporum* f-sp *pisi*, race-6 - occurrence and distribution. Phytopathology 69, 818–820. doi: 10.1094/Phyto-69-818

[ref25] Harling R. Taylor G. S. Matthews P. Arthur A. E. (1988). The effect of temperature on symptom expression and colonization in resistant and susceptible carnation cultivars infected with *fusarium oxysporum* f.sp. *dianthi*. J. Phytopathol. 121, 103–117. doi: 10.1111/j.1439-0434.1988.tb00961.x

[ref26] Ivanovic M. Dragicevic O. Ivanovic D. (1987). *Fusarium oxysporum* f.sp. *fabae* as cause of root rot on broad bean in Yugoslavia. Zastita Bilja 38, 373–380.

[ref27] Jenkins S. Taylor A. Jackson A. C. Armitage A. D. Bates H. J. Mead A.. (2021). Identification and expression of secreted in xylem pathogenicity genes in *fusarium oxysporum* f. sp. *pisi*. Front. Microbiol. 12:593140. doi: 10.3389/fmicb.2021.593140, PMID: 33897626 PMC8062729

[ref28] Jones L. R. (1924). The relation of environment to disease in plants. American J. Bot. 11, 601–609. doi: 10.1002/j.1537-2197.1924.tb05803.x

[ref29] Jones F. R. Linford M. B. (1925). Pea disease survey in Wisconsin. Wisc. Agric. Exp. Sta. Res. Bull. 64:31.

[ref30] Joshi M. Srivastava R. Sharma A. K. Prakash A. (2013). Isolation and characterization of *fusarium oxysporum*, a wilt causing fungus, for its pathogenic and non-pathogenic nature in tomato (*Solanum lycopersicum*). J. Appl. Nat. Sci. 5, 108–117. doi: 10.31018/jans.v5i1.290

[ref31] Kraft J. M. (1994). *Fusarium* wilt of peas (a review). Agron. 14:567.

[ref32] Kraft J. M. Haware M. P. Jiménez-Díaz R. M. Bayaa B. Harrabi M. (1994). Screening techniques and sources of resistance to root rots and wilts in cool season food legumes. Euphytica 73, 27–39. doi: 10.1007/BF00027179

[ref33] Kulkarni S.P. (2006). Studies on *Fusarium oxysporum* Schlecht Fr f.sp. *gladioli* (Massey) Snyd. & Hans. Causing wilt of gladiolus. M.Sc. (Agri.) thesis, University of Agricultural Sciences, Dharwad, India, pp. 245.

[ref34] Landa B. B. Navas-Cortes J. A. Jimenez-Gasco M. D. Katan J. Refig B. Jimenez-Diaz R. M. (2006). Temperature response of chickpea cultivars to races of *fusarium oxysporum* f. sp. *ciceris*, causal agent of fusarium wilt. Plant Dis. 90, 365–374. doi: 10.1094/PD-90-0365, PMID: 30786563

[ref03] Leung H. Nelson R. J. Leach J. E. (1993) “Population structure of plant pathogenic fungi and bacteria,” in Advances in Plant Pathology. eds. Andrews J. H. Tommerup I. C. (London: Academic Press), 157–204.

[ref35] Lin Y. S. (1991). The occurrence of pea wilt and its control in Taiwan. Pl. Prot. Bull. 33, 36–44.

[ref36] Linford M. B. (1928). A *fusarium* wilt of peas in Wisconsin. Wisc. Agric. Exp. Sta. Res. Bull. 85:43.

[ref37] Maheshwari S. K. Gupta J. S. Sharma P. D. (1980). Studies on histopathology of the pathogens associated with wilted and root rotted seeds and plants of pea. Acta Bot. Indica 8, 270–271.

[ref38] Malvick D. K. Percich J. A. (1998). Genotypic and pathogenic diversity among pea- infecting isolates of *Aphanomyces euteiches* from the central and western United States. Phytopathology 88, 915–921. doi: 10.1094/PHYTO.1998.88.9.915, PMID: 18944869

[ref39] Marinangeli C. P. Jones P. J. (2011). Whole and fractionated yellow pea flours reduce fasting insulin and insulin resistance in hypercholesterolaemic and overweight human subjects. Br. J. Nutr. 105, 110–117. doi: 10.1017/S0007114510003156, PMID: 20807459

[ref40] McPhee K. E. Inglis D. A. Gundersen B. Coyne C. J. (2012). Mapping QTL for fusarium wilt race 2 partial resistance in pea (*Pisum sativum*). Plant Breed. 131, 300–306. doi: 10.1111/j.1439-0523.2011.01938.x

[ref41] Naiki T. Morita Y. (1983). The population of spinach wilt fungus, *fusarium oxysporum* f. sp. *spinaciae*, and the wilt incidence in soil. Ann. Phytopathol. Soc. Jpn. 49, 539–544. doi: 10.3186/jjphytopath.49.539

[ref42] Nelson P. E. Burgess L. W. Summerell B. A. (1990). Some morphological and physiological characters of *fusarium* species in section *Liseola* and *Elegans* and similar species. Mycologia 82, 99–106. doi: 10.1080/00275514.1990.12025846

[ref43] Nyvall R. F. Haglund W. A. (1972). Sites of infection of *fusarium oxysporum* f.sp. *pisi* race 5 on peas. Phytopathology 62, 1419–1424. doi: 10.1094/Phyto-62-1419

[ref44] Parihar A. K. Dixit G. P. Bohra A. Gupta D. S. Singh A. K. Kumar N.. (2020) in Genetic advancement in field pea (*Pisum sativum* L.): retrospect and prospect, in accelerated plant breeding. eds. Gosal S. S. Wani S. H. (Cham: Springer), 283–341.

[ref45] Parihar A. K. Dixit G. P. Singh U. Singh A. K. Kumar N. Gupta S. (2021) in Potential of field pea as a nutritionally rich food legume crop, in breeding for enhanced nutrition and bio-active compounds in food legumes. eds. Gupta D. S. Gupta S. Kumar J. (Cham: Springer), 47–82.

[ref46] Parihar A. K. Kumar J. Gupta D. S. Lamichaney A. Naik S. J. S. Singh A. K.. (2022). Genomics enabled breeding strategies for major biotic stresses in pea (*Pisum sativum* L.). Front. Plant Sci. 13:861191. doi: 10.3389/fpls.2022.861191, PMID: 35665148 PMC9158573

[ref47] Pathak V.N. (1984). Laboratory manual of plant pathology. (2nd Edn.). Oxford and IBH Publishing Co. Pvt. Ltd., New Delhi: 11–12.

[ref48] Scott J. C. Gordon T. R. Shaw D. V. Koike S. T. (2010). Effect of temperature on severity of fusarium wilt of lettuce caused by *fusarium oxysporum* f. sp. *lactucae*. Plant Dis. 94, 13–17. doi: 10.1094/PDIS-94-1-0013, PMID: 30754388

[ref49] Sharma A. Sekhon B. S. Sharma S. Kumar R. (2020). Newly isolated intervarietal garden pea (*Pisum sativum* L.) progenies (F7) under north western Himalayan conditions of India. Exp. Agric. 56, 76–87. doi: 10.1017/S0014479719000115

[ref50] Singh P. K. Khan A. Gogoi R. Jaiswal R. K. (2010). Plant leaf extracts and bioagents for eco-friendly management of wilt of pigeonpea caused by *fusarium udum*. Indian Phytopathol. 63, 343–344.

[ref51] Singh J. Nadarajan N. Basu P.S. Srivastava R.P. Kumar L. (2013). Pulses for human health and nutrition. Kanpur: Indian Institute of Pulses Research, 1–35.

[ref52] Snyder W. C. Hansen H. N. (1940). The species concept in *fusarium*. Am. J. Bot. 27, 64–67. doi: 10.1002/j.1537-2197.1940.tb14217.x

[ref53] Somesh S. N. Behera L. Bais R. K. Tiwari A. Kumar S. (2019). Effect of temperature and pH on growth and sporulation of *fusarium oxysporum* f.sp. *lini* (Bolley) Synder and Hensan causing linseed wilt under environmental condition. J. Pharmaco. Phytochem. 8, 1427–1430.

[ref54] Stuthman D. D. Leonard K. J. Miller-Garvin J. (2007). “Breeding crops for durable resistance to disease” in Advances in agronomy. ed. Sparks D. L., vol. 95 (San Diego, CA: Elsevier Academic Press), 319–367.

[ref55] Sukapure R. S. Bhide V. P. Patel M. K. (1957). *Fusarium* wilt of garden peas (Pisum sativum L.) in Bombay state. Indian Phytopathol. 10, 11–17.

[ref56] Thaware D. S. Kohire O. D. Gholve V. M. (2017). Cultural, morphological and molecular variability of *fusarium oxysporum* f.sp. *ciceri* isolates by RAPD method. Int. J. Curr. Microbiol. Appl. Sci. 6, 2721–2734.

[ref57] Webb K. M. Brenner T. Jacobsen B. J. (2015). Temperature effects on the interactions of sugar beet with fusarium yellows caused by *fusarium oxysporum* f. sp. *betae*. Canidian J. Plant Pathol. 37, 353–362. doi: 10.1080/07060661.2015.1071283

[ref58] Wu H. S. Wang Y. Zhang C. Y. Bao W. Ling N. Liu D. Y.. (2009). Growth of in vitro *fusarium oxysporum* f. sp. *niveum* in chemically defined media amended with gallic acid. Biol. Res. 42, 297–304. PMID: 19915738

